# Determination of Biodegradation Potential of *Aspergillus niger*, *Candida albicans*, and *Acremonium sclerotigenum* on Polyethylene, Polyethylene Terephthalate, and Polystyrene Microplastics

**DOI:** 10.1155/2024/7682762

**Published:** 2024-10-28

**Authors:** Ayesha Safdar, Fatima Ismail, Hafsa Iftikhar, Abdul Majid Khokhar, Atika Javed, Muhammad Imran, Bushra Safdar

**Affiliations:** ^1^Department of Biochemistry, The Islamia University of Bahawalpur, Bahawalpur 63100, Punjab, Pakistan; ^2^Department of Biochemistry, Riphah International University, Faisalabad, Pakistan; ^3^Institute for Advanced Study Shenzhen University, Shenzhen 518060, Guangdong, China; ^4^Department of Agronomy, University of Agriculture, Faisalabad 38000, Pakistan

**Keywords:** biodegradation, biofilm formation, FTIR, microplastics, polyethylene, polyethylene terephthalate, polystyrene, SEM

## Abstract

Plastics are used widely in almost every field of life, but their synthetic and persistent nature makes them harmful for the environment. The aim of this research was to evaluate the degradation abilities of *Aspergillus niger*, *Candida albicans*, and *Acremonium sclerotigenum* on microplastics (MPs). MP pieces of 4 ± 1 mm, including polyethylene, polyethylene terephthalate, and polystyrene, were incubated with fungal inoculums for 30 days. The degradation of treated MPs was determined by biofilm formation, weight loss, scanning electron microscopy (SEM), and Fourier transform analyses. The results indicated that the polyethylene MPs treated with *Aspergillus niger* exhibited the highest level of biofilm formation (optical density 1.595) and percentage weight loss (16%). In the case of polyethylene terephthalate and polystyrene MPs, *Acremonium sclerotigenum* and co-culture showed weight loss of 6% and 10%, respectively. *Candida albicans* was observed to be the least effective in biodegradation analyses. SEM observation revealed the surface modifications as holes, pits, cracks, and increased roughness in treated MPs. Fourier transform infrared (FTIR) spectroscopy showed that the chemical structure of each polymer exhibited some variations. The study concluded that the fungal strains play an important role in the biodegradation of plastics and can be utilized to mitigate environmental pollution.

**Summary**

• A novel approach for rapid biofilm formation by fungal strains on MPs.
• The growth of biofilms on the surfaces of MPs influences their degradation.
• An analysis of percentage weight loss can provide a better understanding of biodegradation activity.
• The degradation of polyethylene (PE), polyethylene terephthalate (PET), and polystyrene (PS) alters their functional groups and surface morphologies.
• *Aspergillus niger* (*A. niger*), *Candida albicans* (*C. albicans*), and *Acremonium sclerotigenum* (*A. sclerotigenum*) are proposed as PE, PET, and PS degraders, proving their efficacy in MPs biodegradation.

## 1. Introduction

Plastics are common and slowly biodegrading polymers present in our environment, and in recent years, their use has increased due to a combination of features including ease of production, light weight, flexibility, strength, and low cost [1]. Microplastics (MPs), which are generally defined as pieces of plastic smaller than 5 mm, resulted from the deterioration of large plastic objects, such as those from poorly managed landfills or illegal dumping [2]. MPs, which are widely distributed around the world, are the main cause of plastic contamination in the soil, freshwater, marine, and agroecosystem ecosystems. Additionally, plastics are utilized in agriculture as a covering for pesticides, hormones, fertilizers, and packaging materials, as well as in mulching, low tunnels, and greenhouses [3]. Statistical evaluations have indicated that MPs account for 92.4% of plastic trash and are predominantly composed of PE, PS, polypropylene (PP), and PET [4].

Researchers exploring techniques to reduce MPs pollution have found that MPs are stable in the environment for extended periods of time, but certain microbes still need to break them down. Microbial species exhibit remarkable environmental adaptation, enabling them to breakdown many contaminants, including MPs [5]. The employment of microorganisms to degrade MPs will improve biodegradation while causing no harm to the environment, providing a potential and environmentally friendly approach for facilitating natural bioremediation and influencing the restoration of natural environments without having negative consequences [6]. The process of biodegradation is also influenced by a variety of parameters, including polymer properties, organism type, and pretreatment method. Polymers, particularly plastics, are suitable substrates for heterotrophic microorganisms [7]. Algae, protists, bacteria, and fungi have a tendency to colonize the surfaces of MPs, creating biofilms. Biofilms are made up of a diverse group of microorganisms as well as extracellular polymeric substances (EPS) released by them [8]. *Streptococcus*, *Pseudomonas*, *Staphylococcus*, *Moraxella*, and *Micrococcus*, *Saccharomonospora*, *Actinomycetes* [9], *Aspergillus niger*, *Aspergillus oryzae*, and *Aspergillus flavus* [10], and *Penicillium* and *Acremonium* [11] have been found to have effective biodegradable qualities. *Candida albicans* is also capable of forming biofilm on the surface of MPs [12].

Fungi offer a variety of exceptional ways for dealing with a wide range of complex chemicals, some of which are pollutants and harmful [13]. They have a remarkable capacity for environmental change and can withstand a wide range of contaminants. These contaminants can be broken down and utilized by them for growth or to catalyze significant chemical reactions [14]. Fungi have an adsorptive mode of nutrition, which allows them to take nutrients from their surroundings, even plastic surfaces. Their apical elongation growth strategy allows them to efficiently expand and explore plastic surfaces, boosting their potential to colonize and exploit these materials [15]. The fungal hyphae are capable of secreting digestive enzymes and breaking organic molecules and macromolecules into smaller molecules in order to utilize them. Aerobic conditions release H_2_O and CO_2_, while anaerobic conditions convert them into H_2_O, CO_2_, and CH_4_ [16]. The current study focuses on evaluating the biodegradation abilities of fungal strains, including *Aspergillus niger*, *Candida albicans*, and *Acremonium sclerotigenum*, against PE, PET, and PS MPs.

## 2. Materials and Methods

### 2.1. MPs and Chemicals

PE, PET, and PS were the three types of MPs used in the study. The main sources of MPs in the study were shopping bags (for PE), single-use plastic water bottles (for PET), and disposable food plates (for PS) obtained locally from Bahawalpur, Pakistan. The original materials were cut into square forms with an average side length of 4 ± 1 mm to produce standardized MPs pieces (Table 1). The experiments were conducted using analytical-grade chemicals, substrates, and apparatus provided by the Department of Biochemistry, The Islamia University of Bahawalpur, Pakistan. The polymers were obtained locally from Bahawalpur, Pakistan, and were converted into 4 ± 1 mm pieces.

### 2.2. Fungal Growth and Inoculum Preparation

Previously identified fungal strains, including *A. niger* and *A. sclerotigenum*, were obtained from the Department of Biochemistry, The Islamia University of Bahawalpur, Pakistan [17] and the strain of *C. albicans* was purchased from ATCC (American Type Culture Collection). Fungal strains were grown on Sabouraud Dextrose Agar (SDA) medium plates made by following the culture medium formula recipe: 20 g/L agar, 10 g/L peptone, 40 g/L dextrose, and 0.5 g/L chloramphenicol with a final pH of 5.6 at 25°C. Plates were then inoculated with *A. niger*, *C. albicans*, and *A. sclerotigenum* cultures. Plates containing *A. niger*, *C. albicans*, and *A. sclerotigenum* cultures were incubated for 5 days at 37°C and 30°C, respectively, in an incubator (model FTC 90E, Usmate, Italy). Fungal inoculation was done following the methods described by [18, 19] under aseptic conditions, including sterilization of all apparatus and laminar airflow cabinets with 70% ethanol, wearing personal protective equipment (PPE). A sterilized inoculation loop was used to collect the spores from culture plates that had been incubating for five days and were then transferred into separate 250-mL Erlenmeyer flasks. These flasks contained Sabouraud Dextrose Broth (SDB), prepared with the following medium formula concentrations: 40 g/L dextrose and 10 g/L peptone, with a final pH of 7.0 achieved at 25°C. To facilitate growth and interaction, the flasks, each containing a loop full of separate fungal strains (*A. niger*, *C. albicans*, and *A. sclerotigenum*) assumed to have a similar number of spores, were placed on a rotary shaker (model SI4, Shel Lab, Sheldon). The shaker operated at 120 rpm, and the incubation was carried out for a duration of 72 h at a temperature of 37°C. Subsequently, individual suspensions of the *A. niger*, *C. albicans*, and *A. sclerotigenum* cultures were used in their original form. The co-culture was set up using a combination of these three suspensions after mixing them in equal amounts.

### 2.3. Setup of the Biodegradation Experiment

The biodegradation experiment was divided into three sets, each with a different set of conditions and controls, to evaluate the degradation of three different types of MPs (PE, PET, and PS). The experiment was designed to assess the impact of fungal species (*A. niger*, *C. albicans*, and *A. sclerotigenum*) on the biodegradation of these MPs, both individually and in co-culture. The PE, PET, and PS MPs were washed three times with distilled water after being sterilized using 70% ethanol. MPs biodegradation studies were carried out in presterilized 250-mL Erlenmeyer flasks with all the conditions described in sets 1, 2, and 3. The flasks were then placed in a shaking incubator and incubated for 30 days with continuous shaking at 120 rpm. The control group (negative) included virgin PE, PET, and PS pieces in inoculums devoid of fungus spores. Each experiment was performed in triplicate. The biodegradation study was divided into three sets, as follows.

#### 2.3.1. Set 1: For 30 Days With Continuous Shaking at 120 rpm

(a) 100 mL of negative control (no inoculum) + 0.5 g weight of PE MPs at pH 7.0°C and 37°C; (b) 100 mL of *A. niger* inoculum + 0.5 g weight of PE MPs at pH 7.5°C and 37°C; (c) 100 mL of *C. albicans* inoculum + 0.5 g weight of PE MPs at pH 7.0°C and 37°C; (d) 100 mL of *A. sclerotigenum* inoculum + 0.5 g weight of PE MPs at pH 8.0°C and 37°C; (e) 100 mL of co-culture (*A. niger* + *C. albicans* + *A. sclerotigenum*) + 0.5 g weight of PE MPs at pH 8.0, 37°C.

#### 2.3.2. Set 2: For 30 Days With Continuous Shaking at 120 rpm

(a) 100 mL of negative control (no inoculum) + 0.5 g weight of PET MPs at pH 7.0°C and 37°C; (b) 100 mL of *A. niger* inoculum + 0.5 g weight of PET MPs at pH 7.5°C and 37°C; (c) 100 mL of *C. albicans* inoculum + 0.5 g weight of PET MPs at pH 7.0°C and 37°C; (d) 100 mL of *A. sclerotigenum* inoculum + 0.5 g weight of PET MPs at pH 8.0°C and 37°C; (e) 100 mL of co-culture (*A. niger* + *C. albicans* + *A. sclerotigenum*) + 0.5 g weight of PET MPs at pH 8.0, 37°C.

#### 2.3.3. Set 3: For 30 Days With Continuous Shaking at 120 rpm

(a) 100 mL of negative control (no inoculum) + 0.5 g weight of PS MPs at pH 7.0°C and 37°C; (b) 100 mL of *A. niger* inoculum + 0.5 g weight of PS MPs at pH 7.5°C and 37°C; (c) 100 mL of *C. albicans* inoculum + 0.5 g weight of PS MPs at pH 7.0°C and 37°C; (d) 100 mL of *A. sclerotigenum* inoculum + 0.5 g weight of PS MPs at pH 8.0°C and 37°C; (e) 100 mL of co-culture (*A. niger* + *C. albicans* + *A. sclerotigenum*) + 0.5 g weight of PS MPs at pH 8.0, 37°C.

### 2.4. Quantification of the Biofilms

A biofilm quantification method with crystal violet staining was adopted from [20]. The strong adherence of microbial cells to plastic surfaces makes it impossible to estimate the biofilm that had grown using cell counting or traditional approaches. Thus, the crystal violet assay was used to evaluate the population of microbes on the polymer surface [21]. The biofilm content on each MP surface was quantified using the crystal violet staining method. Four MP pieces were separated from the subsets (a), (b), (c), (d), and (e) of 1, 2, and 3 Sets into sterile petri dishes. All the pieces were washed thoroughly with 2 mL of distilled water, and this step was performed three times. The washed MPs were allowed to dry at room temperature for 45 min 0.5 mL of crystal violet solution (0.1% w/v) was poured into each dish, and the biofilm content on each MP was allowed to stain for 45 min. The extra dye was removed, and the MPs were rinsed three times using 5 mL of distilled water. The MPs were dried completely at room temperature before being transferred into centrifuge tubes with 1 mL of 95% (v/v) ethanol. All the stained pieces of MPs were allowed to decolorize for 10 min. The decolorizing solution was transferred into a cuvette, and a spectrophotometer (model LABINDIA UV-3000+) at 595 nm was used to measure the optical density, which reflects the quantity of biofilm produced on the surface of each MP. A negative control was obtained using the same method, and it consisted of decolorizing solution alone (95% ethanol solution) with virgin MP pieces. All the experiments were performed in triplicates to ensure the reliability of the results.

### 2.5. Weight Loss Assessment of MPs

The MPs were removed from each treatment and controls after 30 days of incubation. An analytical balance (model Shimadzu AUX-320) was utilized to weigh the MPs, which had been previously measured in triplicate. Equation (1) was used to obtain the percentage weight reduction (%) across all treatments [22]:

(1) $$\begin{array}{c} \%\text{ Weight loss}=100\frac{\left(W_{0}–W_{t}\right)}{W_{0}}, \end{array}$$

where *W*_0_ denotes the initial weight of MPs (in grams) prior to biodegradation. *W*_t_ denotes the final weight of MPs (in grams) after biodegradation.

### 2.6. SEM Analysis for Morphological Study

The surface morphologies of MPs both before and after the biodegradation experiment were analyzed using a scanning electron microscope (Hitachi S2380N SEM) located in the Central Hi-Tech Laboratory, University of Agriculture, Faisalabad, Pakistan. For this purpose, PE, PET, and PS MPs treated separately with *A. niger*, *C. albicans*, *A. sclerotigenum*, and co-cultures, were washed with 70% ethanol and dried completely. The dried and gold-coated MP samples and negative controls were analyzed under Hitachi S2380N SEM at 1.8 kV. The micrographs for both controls and treated samples were then captured.

### 2.7. FTIR Methods for Functional Group Determination

The structural modifications in all MPs were examined using an Agilent Cary 630-FTIR spectrometer at the Department of Chemistry, The Islamia University of Bahawalpur, Pakistan. PE, PET, and PS MPs treated with fugal inoculums for 30 days were examined. The noise in the background was removed by running a blank scan in the 4000 − 650 cm^−1^ frequency range. The resulting spectrum contained a graph depicting transmittance rate against wave length (2.5–15 μm) within the frequencies ranging from 4000 to 650 cm^−1^.

### 2.8. Statistical Methods

All experiments were conducted in triplicate (*n* = 3), with negative controls not treated with fungal inoculums. The results were presented as *x̄* ± SE (mean ± standard error). To ascertain the group means, an analysis of variance (ANOVA) was conducted, and Tukey's HSD (honestly significant difference) test was performed after the comparison of these means. Statistical significance was set at *p* < 0.05. For the statistical analysis, the software Statistics 8.1 was employed, and the graphs were generated using Origin 8.0 and Microsoft Excel.

## 3. Results

**Figure 1:**
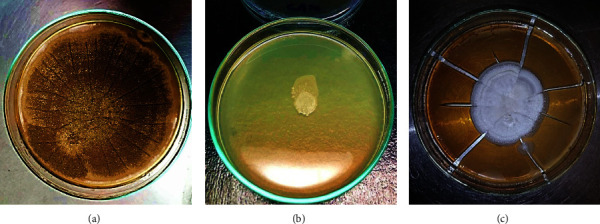
The colony of grown fungal strains after 5 days of incubation on SDA plates. (a) *A. niger* growth; (b) *C. albicans* growth; (c) *A. sclerotigenum* growth.

The primary goal of this study is to focus on fungal strains (*A. niger*, *C. albicans*, and *A. sclerotigenum*) for MP degradation because research on the degradation of MPs by fungi is limited and requires more progress. Out of three fungi, *A. niger* (accession number MG654699.1) and *A. sclerotigenum* (accession number MK732096.1) were isolated and identified from our earlier research [17], while *C. albicans* (accession number ATCC10231) was purchased from ATCC. All of these were grown on the SDA media plates, as shown in Figure 1. The study evaluated the biodegradability potential of the selected fungi and their ability to utilize different polymers (PE, PET, and PS MPs). Fungal biofilms have the ability to expand across substrates in search of nutrients and develop in regions difficult to access by other microbes. The outer sides of fungal hyphae excrete digestive enzymes through exocytosis and convert the polymers into monomers, dimers, and oligomers. The quantitative study of biofilm development, measured percentage weight loss, SEM, and FTIR analysis were used to evaluate the ability of these isolates to significantly enhance the biodegradation of MPs within 30 days.

### 3.1. Quantitative Analysis of Biofilms

**Figure 2:**
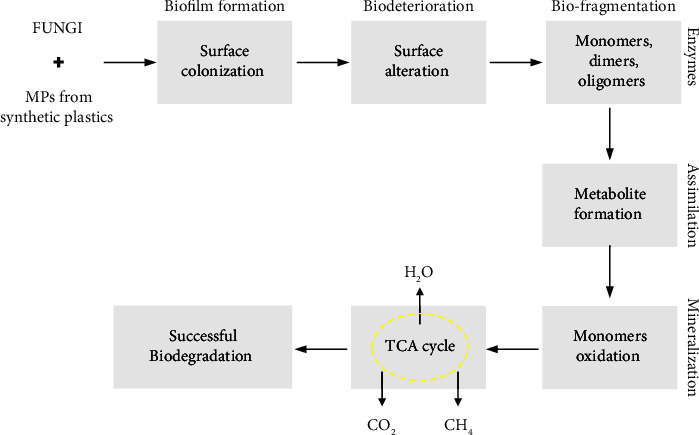
The mechanism of plastic degradation by fungi. In the beginning, fungi capable of degrading plastics attach to the surface irreversibly and form biofilms. In the next stage, fungal biofilms release extracellular enzymes, convert the long chains of polymers into monomers, dimers, and oligomers and start the surface alteration phenomenon. Fungal strains used in degradation pathways assimilate these monomers, dimers, and oligomers through the citric acid or TCA (tricarboxylic acid) cycle. Finally, H_2_O, CO_2_, and CH_4_ are produced after the mineralization of monomeric and oligomeric units depending on aerobic or anaerobic conditions.

The total amount of biofilm generated on PE, PET, and PS MP surfaces was quantified using the method of crystal violet staining. During the 30-day incubation period, the total amount of biofilm on the PE surface exposed to *A. niger* inoculum was significantly higher than the others (*p* < 0.05). According to statistical analysis, PE in the *A. niger* inoculum had a greater impact on fungal colonization (biofilm) than any other condition under biodegradation.  Table 2 shows that all the values with different letters demonstrated statistical significance (*p* < 0.05). The major mechanism behind the formation of fungal biofilms and polymeric degradation is shown in Figure 2. The main steps involved in these mechanisms were biofilm formation, biodeterioration, biofragmentation, assimilation, and mineralization. All these processes converted the large polymeric units into smaller ones to be utilized for successful biodegradation.

### 3.2. Weight Loss Analysis of MPs

The alterations in total weight of MPs were determined by calculating the weight loss of PE, PET, and PS MPs. The results after statistical analysis are outlined in Table 3. The percentage weight loss for PE MPs within *A. niger*, *C. albicans*, *A. sclerotigenum*, and co-culture was 16%, 5%, 6%, and 3.8%, respectively. The maximum weight loss was noted during PE degradation when treated with the *A. niger* inoculum. In the case of PET MPs, weight loss measured with *A. niger*, *C. albicans*, *A. sclerotigenum*, and co-culture was 3%, 2%, 4.6%, and 4.2%, respectively. PS MPs exhibited 4%, 2.2%, 8%, and 10% of weight loss when compared to control. *A. sclerotigenum* and co-cultures recorded a percentage weight loss of 8%, 6%, 4.6%, and 10%, 3.8%, and 4.2% for PS, PE, and PET MPs, respectively. The minimum weight loss noted was with *C. albicans*, i.e., 5%, 2.2%, and 2% for PE, PS, and PET MPs, respectively. The results suggested that the fungal strains had the capacity to excrete particular enzymes that could potentially target these MPs, leading to their biodegradation. The isolated strains of *A. niger*, *C. albicans*, and *A. sclerotigenum* might have facilitated the metabolic processes that played a role in the adsorption, desorption, and degradation of PE, PET, and PS MPs.

### 3.3. SEM Analysis of Biodegraded MPs

**Figure 3:**
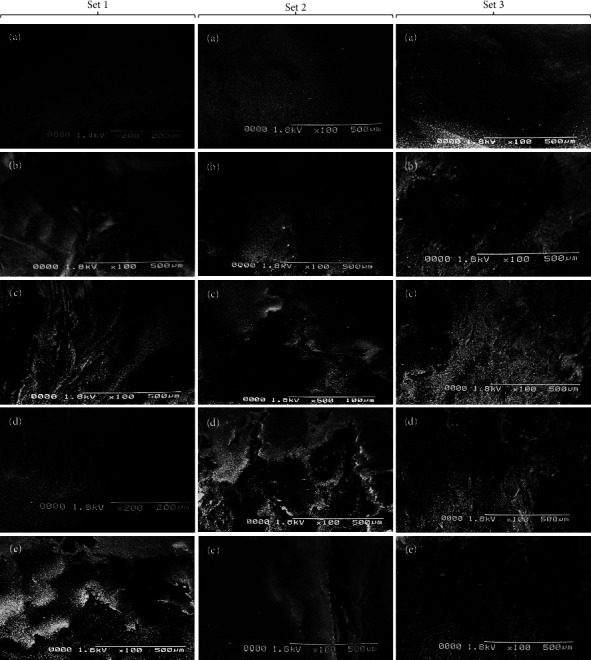
SEM images of three microplastics after 30 days of incubation. Set 1: (a) PE + control; (b) PE + *A. niger*; (c) PE + *C. albicans*; (d) PE + *A. sclerotigenum*; (e) PE + co-culture (*A. niger* + *C. albicans* + *A. sclerotigenum*). Set 2: (a) PET + control; (b) PET + *A. niger*; (c) PET + *C. albicans*; (d) PET + *A. sclerotigenum*; (e) PET + co-culture (*A. niger* + *C. albicans* + *A. sclerotigenum*). Set 3: (a) PS + control; (b) PS + *A. niger*; (c) PS + *C. albicans*; (d) PS + *A. sclerotigenum*; (e) PS + co-culture (*A. niger* + *C. albicans* + *A. sclerotigenum*).

A remarkable electron microscopic technique known as the SEM is renowned for its ability to generate a detailed, vivid image of an object with exceptional clarity and precision. SEM is a useful tool for characterizing materials and is frequently employed to investigate surface-related phenomena in material samples [23]. SEM of fungus-treated samples showed the biodegradation of PE, PET, and PS in comparison to the controls (un-inoculated MPs). The un-inoculated MPs showed smooth surfaces without any cracks, fissures, or holes. The surface of MPs that have undergone microbial treatment displayed a variety of grooves, fissures, pores, and cracks, supporting the potential of fungal biofilms to degrade these MPs. Scanning electron images of PE, PET, and PS MPs in controlled and inoculated samples are shown in Figure 3. The initial morphologies of MPs changed from smooth to irregular surfaces during the process of biodegradation. The fungal strains utilized in this study caused fragility, cracks, grooves, and surface anomalies in treated MPs. As per the photomicrographs biodegradation of PE MPs by using *A. niger* (Figure 3(b) of Set 1), *C. albicans* (Figure 3(c) of Set 1), and co-culture (*A. niger* + *C. albicans* + *A. sclerotigenum*) as shown in Figure 3(e) of Set 1 showed promising results. All these treated MPs showed cracks and irregular surfaces when compared to the un-inoculated MPs (Figure 3(a) of Set 1). Figure 3(d) of Set 1 exhibits the formation of grooves and holes in the PE surface after the treatment with *A. sclerotigenum*. In the case of PET and PS MPs, *A. sclerotigenum* and co-culture (*A. niger* + *C. albicans* + *A. sclerotigenum*) showed maximal biodegradation, as shown in Figure 3(d) of Set 2 and Figure 3(e) of Set 3. The PET surface in this case showed cracks, grooves, and fissures, while the PS surface was destroyed completely. Figures 3(c) and 3(e) of Set 2 presented the creation of cracks and fissures, while Figure 3(b) of Set 2 showed the pits and holes in comparison to the control's smooth surface (Figure 3(a) of Set 2). Figure 3(a) of Set 3 shows the smooth surface of PS control, while Figure 3(b) of Set 3 shows an irregular and rough surface. Figures3(c) and 3(d) of Set 3 also showed surface deformities when compared to the PS control (Figure 3(a) of Set 3). This proved that treated strains played a part during the biodegradation pathway, as cracks, pits, and grooves could be seen on MP surfaces. Once attached to the surface of MPs, the microbes in the biofilms release EPS and extracellular enzymes, leading to the destruction of the MPs.

### 3.4. FTIR Analysis of MPs

**Figure 4:**
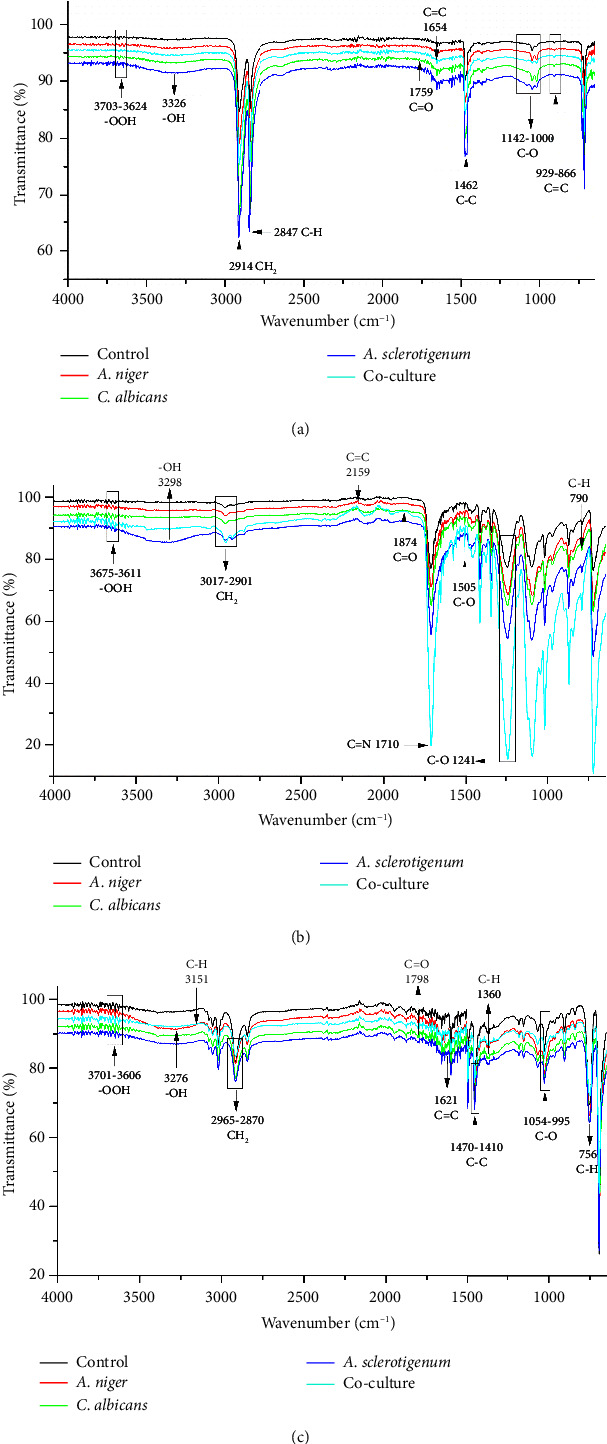
FTIR analysis shows the chemical transformation of fungus-treated and control samples in (a) PE, (b) PET, and (c) PS after 30 days of biodegradation. Graph lines in black (control), red (*A. niger*), green (*C. albicans*), dark blue (*A. sclerotigenum*), and light blue (co-culture) in (a–c).

FTIR is a sensitive and fast analytical instrument that can detect any modification or alteration in the functional groups that are present in polymeric materials [24]. While the percentage of weight loss was noted, it was equally important to determine how the presence of fungal species changed the chemical composition of the MPs. In order to determine the changes in PE, PET, and PS MPs in controls, *A. niger*, *C. albicans*, *A. sclerotigenum* inoculums, and co-cultures, FTIR-ATR studies were performed, as shown in Figure 4. Alterations in bond cleavage, chemical conversions, and the generation of new functional groups have all been noticed during the biodegradation of polymers. The alterations to bonds like C-C, C-O, C=O, C-H, and C=C and the inclusion of hydroxyl, carboxyl, and phenol groups were noticed during the process of biodegradation. The results showed the emergence of new, sharp peaks linked to C-O (alkoxy groups) in PE MPs at 1000–1142 cm^−1^ (Figure 4(a)), in PET MPs at 1241 cm^−1^ (Figure 4(b)), and in PS MPs at 995–1054 cm^−1^ (Figure 4(c)). The peaks attributing to C=O (carbonyl bonds) were noted in *C. albicans*-treated PE and PS MPs and co-culture treated PET MPs at 1759 cm^−1^, 1798 cm^−1^, and 1874 cm^−1^, respectively.

The C=C (alkene stretches) were formed in all PE MPs at 866–929 cm^−1^, as shown in Figure 4(a). The C=C stretches were also formed in co-culture-treated PE (at 1654 cm^−1^), *A. niger*-treated PET (at 2159 cm^−1^), and *A. sclerotigenum*-treated PS (at 1621 cm^−1^) MPs. A stretch attributing C=N at 1710 cm^−1^ was observed only in co-culture-treated PET MPs in comparison to others. The peaks specific to C-C emerged at 1462 cm^−1^ in *A. sclerotigenum*-treated PE, at 1505 cm^−1^ in co-culture-treated PET, and at 1410–1470 cm^−1^ in PS samples. The emergence of C–H bonds at multiple locations (Figures 4(a), 4(b), 4(c)) subsequent to treatment with fungal strains was also noted, providing confirmation of structural modifications resulting from the process of biodegradation. Moreover, the expanded peaks around 3200–3700 cm^−1^ of -OH and -OOH bonding associated to hydroxyl, phenol, or carboxyl groups and the intense distinctive peaks because of C-H bending and stretching show the successful achievement of surface modification. It is believed that the formation of various hydrolyzing bonds (carbonyl, alkoxy, hydroxyl, and alkene) in MPs was a crucial step in the biodegradation process. The current work was facilitated by utilizing the fungal strains (*A. niger*, *C. albicans*, and *A. sclerotigenum*) to alter these functional groups and chemical bonds. Without the presence of these functional groups, there would be no interaction between the fungal strains and the MPs samples. Compared to the control groups, the peak intensities in the other spectra showed changes, suggesting that the fungal strains were actively involved in the degradation of MPs.

## 4. Discussion

In recent years, a rising number of microbes capable of degrading MPs in the laboratory have been discovered. Fungi are a wide and complex community of eukaryotic organisms classified morphologically as yeasts, dimorphic, or filamentous fungi. According to some research, fungi are generally superior degraders of polyurethane and PE to bacteria [25]. Our results indicated that the highest biodegradation rate of 16% was shown by *Aspergillus niger* when treated with PE MPs, while the least was noted with *Candida albicans*. Biodegradation is based on the growth of a biofilm, which is a thin layer formed by microorganisms attaching to the surface of the polymer with their produced polysaccharides. The polymer is then degraded into smaller molecular units, which is likely aided by enzymes produced by the microbes. The extracellular enzymes of microorganisms are able to attack the C-C backbone of PE and PS or to cleave the ester backbone of PET. Microbial metabolism in biofilm is essential for the mineralization of both inorganic and organic substances, as well as subsequent biodegradation. The microorganisms rapidly take up and digest these degraded metabolites. These small molecules are then used by microbes as energy and carbon sources [26]. Several studies have also proven that fungi can break down polymers. The interaction of *Acremonium strictum* strain KR21-2 and PS MPs resulted in the formation of biofilms on MP surfaces [27]. Zhang et al. employed the PE-degrading fungus *Aspergillus flavus*, designated as PEDX3. After 28-day exposure to the PEDX3 strain, a mass loss percentage of 3.9025 ± 1.18% was recorded [28]. The application of *Bacillus subtilis*, *Bacillus pumilus*, and *Kocuria palustris* for PE degradation resulted in 1.75%, 1.5%, and 1% weight loss, respectively [29]. In the present study, the biodegradation was analyzed based on data that has already been published [30, 31]. Our findings support earlier research showing that microbial populations may colonize the polymer surface and cause degradation of that surface.

The SEM results of the present work showed that the presence of biofilm caused MPs to develop pits, grooves, cracks, and fragility on the polymer surface. The roughness of MP surfaces was also increased by *A. niger*, *C. albicans*, and *A. sclerotigenum* biofilms. This was basically due to the fact that fungi used these polymers as substrates, and biofilms affected the fate of MPs by changing their physical characteristics and participating in their disintegration. Two fungal strains, *Penicillium chrysogenum* NS10 and *Penicillium oxalicum* NS4 were also reported to cause structural alterations such as pits, grooves, cracks, roughening, and fragility of the PE surface [32]. The treatment of PET film with *Aspergillus niger* (STF2) and *Aspergillus flavus* (STF1) resulted in the appearance of multiple grooves, cracks, and fissures after a 60-day incubation period [33]. Thus, our findings support prior research indicating fungal strains have the ability to adhere to the surface of MPs, causing their erosion and degradation. The FTIR studies of the current work confirmed that the shifts in peak patterns were caused by the metabolic processes of the *A. niger*, *C. albicans*, and *A. sclerotigenum* consortiums. The strains also involved oxidation-reduction reactions occurring on the MP surface during the 30-day incubation period. In a study conducted by Elsamahy et al. comparable shifts in peak patterns were noted within their investigation of PE degradation using a fungal consortium [34]. FTIR analysis of biologically treated PE with *Aspergillus* spp. showed the emergence of alkanes, alkenes, amines, and alcohols [35]. Another study used *Pseudomonas, Penicillium*, and *Aspergillus* spp. for PS, PE, and PET degradation and found the formation of alkene, carbonyl, alkoxy, and hydroxyl functional groups using FTIR analysis [21]. Tareen et al. (2022) observed identical biodegradation results using FTIR analysis for functional group alterations, consistent with the current study findings [31]. Our findings from FTIR are also in accordance with [36]. The utilization of FTIR analysis unveiled the emergence of distinct functional groups as compared to the control, confirming the ability of fungal strains for polymer degradation throughout the depolymerization procedure.

Over 400 kinds of microbes have been found for plastic degradation. However, there is still tremendous room for improvement in MP biodegradation, notably in terms of the efficacy of various foreign microbes in breaking down plastics [37]. Researchers are particularly interested in *Aspergillus* species because of their widespread distribution, simple isolation, and possible capacity to degrade plastic and polymeric materials [38, 39]. Furthermore, *Aspergillus* is superior to several bacteria from *Brevibacillus*, *Bacillus*, *Cellulosimicrobium*, *Pseudomonas*, *Lysinibacillus*, or *Ochrobactrum* in their ability to degrade PE [40]. The current study primarily focused on characterizing MP following biodegradation. The results obtained in all lab-established experiments, including biofilm formation, weight loss, SEM, and FTIR visualization, are in agreement with each other. The highest amount of biofilm was observed on the PE surface when exposed to *A. niger*, followed by PS in co-culture and *A. sclerotigenum*. The weight loss, SEM, and FTIR analyses also confirmed the maximal changes in weights and physical and chemical structures of the *A. niger*-treated PE MPS, followed by co-culture and *A. sclerotigenum*-treated PS MPs. In short, fungi can break down complex polymers, making them effective bioremediation agents. Several factors may influence the toxic effects of pollutants, including changes in their shape, chemical properties, coexistence with microorganisms, and the addition of other pollutants. Despite the biodegradation of MP by fungal strains showing promise for reducing plastic pollution, a thorough knowledge of the underlying metabolites, genes, and proteins remains unclear. Future research should focus on the underlying processes of polymer degradation by fungal species using metagenomic analysis, investigating gene expression patterns under various environmental conditions, and acquiring extensive knowledge of the involved metabolites, genes, and proteins. By expanding our expertise in this field, we can open the road for effective solutions to reduce plastic pollution.

## 5. Conclusion

Pollution has become a severe concern in recent years, and a safe and environmentally beneficial method known as biodegradation is required for the fate of plastic waste. The findings of this study indicated that *A. niger*, *C. albicans*, and *A. sclerotigenum* have the ability to degrade three MPs (PE, PET, and PS). The formation of biofilms on treated MPs exhibited weight loss and variations in their surface and functional characteristics. Treatment of *A. niger* on PE MPs showed maximal optical density and percentage weight loss, followed by co-culture and *A. sclerotigenum* treatment with PS MPs, respectively. SEM and FTIR analyses confirmed that fungus-treated samples showed holes, pits, fissures, cracks, and alterations of various functional groups, including hydroxyl, carbonyl, phenol, alkoxy, carboxyl, and alkene. The study found that fungal strains play an important role in degrading synthetic plastics and may offer a viable solution for environments contaminated with plastic materials.

## Figures and Tables

**Table 1:** Microplastics selected for treatment under different conditions.

Polymer type	Abbreviation	Size (mm)	Density (g/cm^−3^)	Color	Shape	Source	Physical properties
Polyethylene	PE	4 ± 1	0.94	White-grey	Square fragments	Shopping bag	Thin, strong, flexible, and water proof
Polyethylene terephthalate	PET	4 ± 1	1.35	Transparent-white	Square fragments	Single-use plastic water bottle	Lightweight, shatter proof, and strong
Polystyrene	PS	4 ± 1	0.98	White	Square fragments	Disposable food plate	Inert, smooth, soft, and heat resistant

**Table 2:** Biofilm formation on PE, PET, and PS microplastics by the inoculations under varied treatment conditions.

Treatment	Biofilm formation on microplastics as measured by optical density (OD) at 595 nm		
	PE	PET	PS
Control	0.155 ± 0.000^k^	0.155 ± 0.000^k^	0.155 ± 0.000^k^
*A. niger*	1.595 ± 0.001^a^	0.578 ± 0.004^h^	0.709 ± 0.001^f^
*C. albicans*	0.660 ± 0.010^g^	0.271 ± 0.001^j^	0.520 ± 0.012^i^
*A. sclerotigenum*	0.775 ± 0.001^e^	0.920 ± 0.012^d^	1.135 ± 0.003^c^
Co-culture	0.733 ± 0.006^f^	0.797 ± 0.002^e^	1.171 ± 0.007^b^

*Note:* The values given are the mean with +SEM (*n* = 03). Means denoted by different letters are statistically significant at 0.05 level of significance.

**Table 3:** Analysis of weight loss in PE, PET, and PS microplastics under varied treatment conditions.

Treatment	Weight loss analysis of microplastics under varied treatments (after a 30-day incubation period)		
	PE	PET	PS
Control	0.0 ± 0.000^k^	0.0 ± 0.000^k^	0.0 ± 0.000^k^
*A. niger*	16 ± 0.060^a^	3.0 ± 0.062^i^	4.0 ± 0.070^gh^
*C. albicans*	5.0 ± 0.115^e^	2.0 ± 0.064^j^	2.2 ± 0.063^j^
*A. sclerotigenum*	6.0 ± 0.173^d^	4.6 ± 0.058^f^	8.0 ± 0.058^c^
Co-culture	3.8 ± 0.058^h^	4.2 ± 0.057^g^	10 ± 0.064^b^

*Note:* The means denoted by different letters are statistically different at the 0.05 level of significance. *n* = 03.

## Data Availability

The data used to support the findings of this study are available from the corresponding author on reasonable request.

## Nomenclature

PE: Polyethylene
PET: Polyethylene terephthalate
PS: Polystyrene
MPs: Microplastics
*A. niger*: *Aspergillus niger*
*C. albicans*: *Candida albicans*
*A. sclerotigenum*: *Acremonium sclerotigenum*
SEM: Scanning electron microscopy
EPS: Extracellular polymeric substances
FTIR: Fourier transform infrared spectroscopy.
